# Low lymphocyte count and high monocyte count predicts poor prognosis of gastric cancer

**DOI:** 10.1186/s12876-018-0877-9

**Published:** 2018-10-11

**Authors:** Fan Feng, Gaozan Zheng, Qiao Wang, Shushang Liu, Zhen Liu, Guanghui Xu, Fei Wang, Man Guo, Xiao Lian, Hongwei Zhang

**Affiliations:** 1 Division of Digestive Surgery, Xijing Hospital of Digestive Diseases, Fourth Military Medical University, 127 West Changle Road, Xi’an, 710032 Shaanxi China; 2 Department of General Surgery, No. 91 Hospital of PLA, Jiaozuo, 454000 Henan China; 3 Department of General Surgery, No. 534 Hospital of PLA, Luoyang, 471000 Henan China

**Keywords:** Gastric cancer, Lymphocyte, Monocyte, Prognosis

## Abstract

**Background:**

Existing data about the prognostic value of absolute count of blood cells in gastric cancer was limited. Thus, the present study aims to investigate the prognostic value of absolute count of white blood cell (WBC), neutrophil, lymphocyte, monocyte and platelet in gastric cancer patients.

**Methods:**

From September 2008 to March 2015, 3243 patients treated with radical gastrectomy were enrolled in the present study. Clinicopathological characteristics were recorded. The prognostic value of blood test in gastric cancer patients were analyzed.

**Results:**

There were 2538 male and 705 female. The median age was 58 years (range 20–90). The median follow-up time was 24.9 months (range 1–75). The 1-, 3- and 5-year overall survival rate was 88.9%, 65.8% and 57.2%, respectively. The optimal cut off value was 6.19 × 109/L for WBC (*P* = 0.146), 4.19 × 109/L for neutrophil (*P* = 0.004), 1.72 × 109/L for lymphocyte (*P* = 0.000), 0.51 × 109/L for monocyte (*P* = 0.019) and 260.0 × 109/L for platelet (*P* = 0.002), respectively. Neutrophil, lymphocyte, monocyte and platelet were risk factors for the prognosis of gastric cancer (all *P* < 0.05). However, only lymphocyte and monocyte were independent risk factors (both *P* < 0.05). Combination of lymphocyte and monocyte could increase the prognostic value for gastric cancer patients, especially in stage II/III gastric cancer patients.

**Conclusions:**

High absolute count of neutrophil, monocyte and platelet, and low absolute count of lymphocyte were associated with poor prognosis of gastric cancer. However, only lymphocyte and monocyte count were independent prognostic predictors. Combination of lymphocyte and monocyte count could further increase the predictive value for gastric cancer.

## Background

Up to date, the prognosis and treatment of gastric cancer patients after radical gastrectomy mainly depends on TNM stage system. However, the prognosis of gastric cancer patients could be various even with the same tumor stage. Thus, additional parameters need to be defined to better evaluate the prognosis of patients.

Over the past decades, prognostic value of blood test parameters in gastric cancer patients has been investigated by numerous studies [1, 2]. Because blood test is simple, convenient, reproducible and cost-effective. However, the most common parameters been investigated in the previous reports were neutrophil-to-lymphocyte ratio (NLR) and platelet-to-lymphocyte ratio (PLR) [3, 4]. As NLR and PLR could effectively reflect the inflammation and immune status in vivo, which have been demonstrated to be associated with the progression and prognosis of tumors. However, the prognostic value of absolute count of blood cells, which was more convenient, has rarely been investigated in gastric cancer patients.

Given this situation, the present study aims to investigate the prognostic value of absolute count of WBC, neutrophil, lymphocyte, monocyte and platelet in gastric cancer patients.

## Methods

This study was performed in the Division of Digestive Surgery, Xijing Hospital of Digestive Diseases. From September 2008 to March 2015, a total of 3243 gastric cancer patients was enrolled in the present study. All patients were treated with radical D2 gastrectomy and regular follow up. This study was approved by the Ethics Committee of Xijing Hospital, and written informed consent was obtained from all patients before surgery.

Preoperative blood test was performed within 7 days before surgery. Absolute count of WBC, neutrophil, lymphocyte, monocyte and platelet were recorded. Patients with signs of infection were excluded. Clinicopathological data including gender, age, tumor location, tumor size, pathological type, tumor depth, lymph node metastasis and tumor stage were collected. The patients were followed up till November 2015 every 3 months.

Data were processed using SPSS 22.0 for Windows (SPSS Inc., Chicago, IL, USA). The best cut off value of absolute count of WBC, neutrophil, lymphocyte, monocyte and platelet for the prognosis of gastric cancer were calculated using X-tile software. X-tile is a statistical software cut-point selection. The X-tile software allows the user to move a cursor across the grid and provides an “on-the-fly” histogram of the resulting population subsets along with an associated Kaplan-Meier curve [5]. Risk factors for the prognosis of gastric cancer identified by univariate analysis were further assessed by multivariate analysis using the Cox’s proportional hazards regression model. Overall survival was analyzed by Kaplan-Meier method. The *P* value was considered to be statistically significant at 5% level.

## Results

The clinicopathological characteristics of the entire cohort was summarized in Table 1. There were 2538 male and 705 female. The median age was 58 years (range 20–90). The median follow-up time was 24.9 months (range 1–75). The 1-, 3- and 5-year overall survival rate was 88.9%, 65.8% and 57.2%, respectively.

**Table 1:** Clinicopathological characteristics of patients

Characteristics	*n* = 3243
Gender	
Male	2538
Female	705
Age	
≤ 60	1930
> 60	1313
Tumor location	
Upper third	1022
Middle third	531
Lower third	1429
Entire	261
Tumor size (cm)	
≤ 5	2248
> 5	995
Pathological type	
Well differentiated	365
Moderately differentiated	827
Poorly differentiated	1866
Signet ring cell or Mucinous	185
Tumor depth	
T1	603
T2	499
T3	1165
T4	976
Lymph node metastasis	
N0	1162
N1	623
N2	562
N3	896
Tumor stage	
I	801
II	946
III	1496
WBC (10^9^/L)	5.82 ± 1.47
Neutrophil (10^9^/L)	3.62 ± 1.29
Lymphocyte (10^9^/L)	1.68 ± 0.56
Monocyte (10^9^/L)	0.39 ± 0.16
Platelet (10^9^/L)	215.81 ± 77.53

**Fig. 1:**
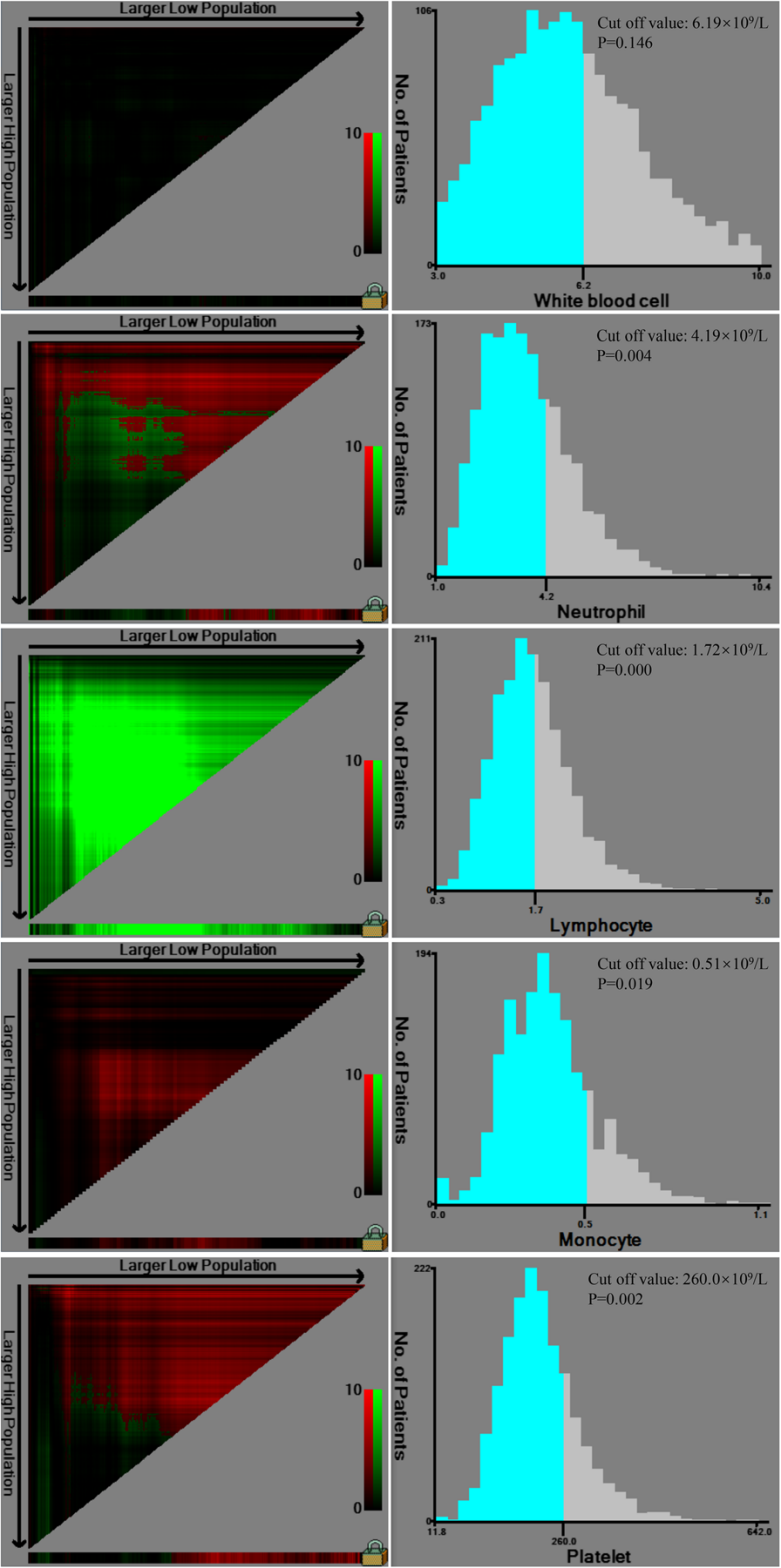
Calculation of cut off value of WBC, neutrophil, lymphocyte, monocyte and platelet count by X-tile software

The optimal cut off value of absolute count of WBC, neutrophil, lymphocyte, monocyte and platelet for the prognosis of gastric cancer patients were shown in Fig. 1. The optimal cut off value was 6.19 × 10^9^/L for WBC (*P* = 0.146), 4.19 × 10^9^/L for neutrophil (*P* = 0.004), 1.72 × 10^9^/L for lymphocyte (*P* = 0.000), 0.51 × 10^9^/L for monocyte (*P* = 0.019) and 260.0 × 10^9^/L for platelet (*P* = 0.002), respectively.

**Fig. 2:**
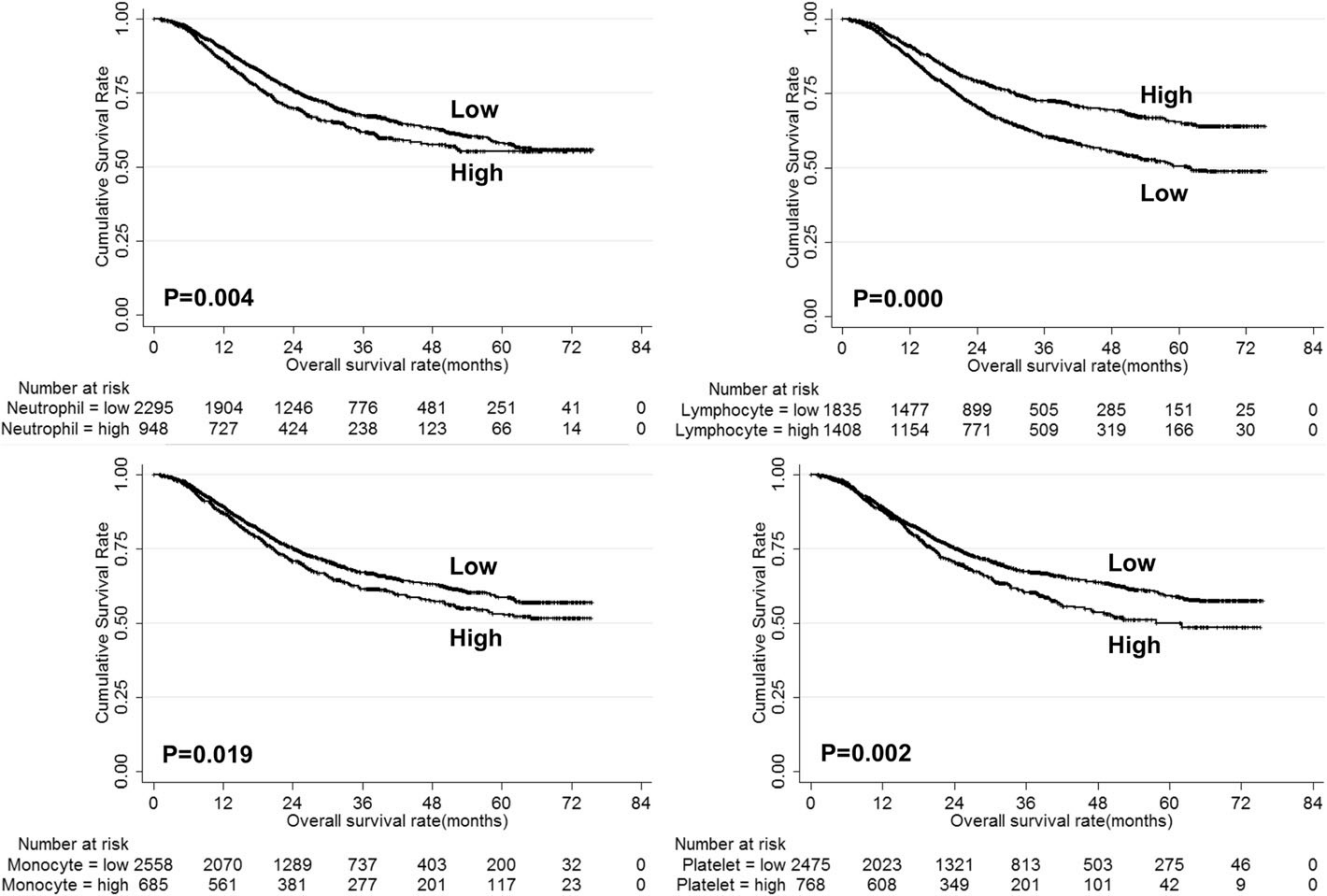
Overall survival of gastric cancer patients according to neutrophil, lymphocyte, monocyte and platelet count

**Fig. 3:**
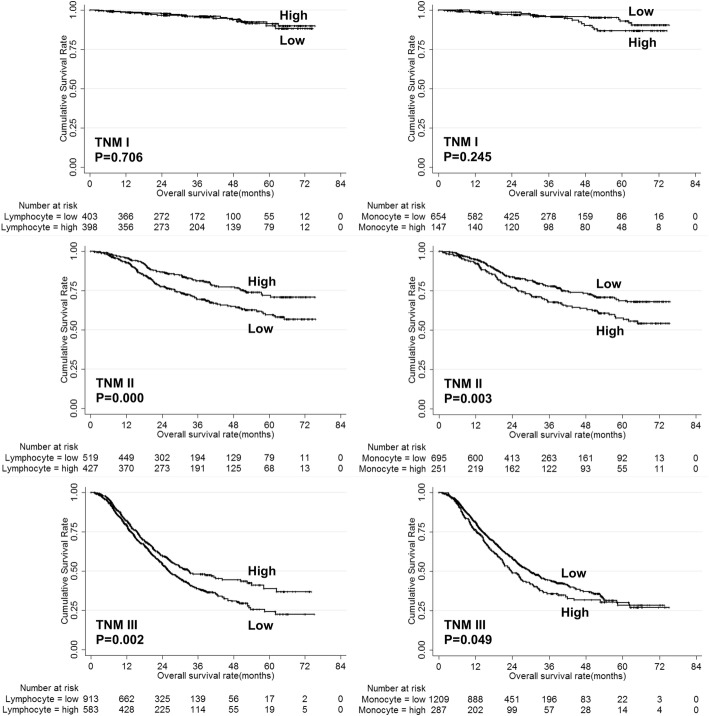
Overall survival according to lymphocyte and monocyte count stratified by TNM stage

The univariate analysis showed that age, tumor size, pathological type, tumor depth, lymph node metastasis, tumor stage, neutrophil, lymphocyte, monocyte and platelet were risk factors for the prognosis of gastric cancer (Table 2). The overall survival of gastric cancer patients according to neutrophil, lymphocyte, monocyte and platelet were shown in Fig. 2. However, only age, tumor size, tumor depth, lymph node metastasis, lymphocyte and monocyte were independent risk factors for the prognosis of gastric cancer (Table 2). Then, the prognostic value of lymphocyte and monocyte were analyzed stratified by TNM stage. The results showed that both lymphocyte and monocyte could predict the prognosis of stage II/III gastric cancer patients (Fig. 3).

**Table 2:** Univariate and multivariate analysis of risk factors for prognosis of gastric cancer patients

Prognostic factors	Univariate			Multivariate		
	β	HR (95% CI)	P	β	HR (95% CI)	P
Gender	0.072	1.075 (0.922–1.253)	0.354			
Age	0.258	1.294 (1.139–1.470)	0.000	0.258	1.295 (1.137–1.474)	0.000
Tumor location	−0.030	0.971 (0.910–1.035)	0.363			
Tumor size	1.105	3.020 (2.656–3.433)	0.000	0.410	1.507 (1.313–1.731)	0.000
Pathological type	0.433	1.541 (1.414–1.681)	0.000	0.093	1.098 (0.996–1.210)	0.060
Tumor depth	0.795	2.214 (2.043–2.398)	0.000	0.440	1.553 (1.416–1.703)	0.000
Lymph node metastasis	0.710	2.034 (1.917–2.159)	0.000	0.503	1.653 (1.544–1.770)	0.000
Tumor stage	1.239	3.452 (3.082–3.867)	0.000			
White blood cell	0.098	1.102 (0.966–1.258)	0.146			
Neutrophil	0.205	1.227 (1.068–1.409)	0.004	0.038	1.038 (0.897–1.202)	0.613
Lymphocyte	−0.433	0.648 (0.567–0.741)	0.000	−0.254	0.776 (0.677–0.888)	0.000
Monocyte	0.173	1.189 (1.029–1.374)	0.019	0.257	1.293 (1.111–1.505)	0.001
Platelet	0.232	1.262 (1.091–1.459)	0.002	−0.096	0.908 (0.781–1.056)	0.211

**Fig. 4:**
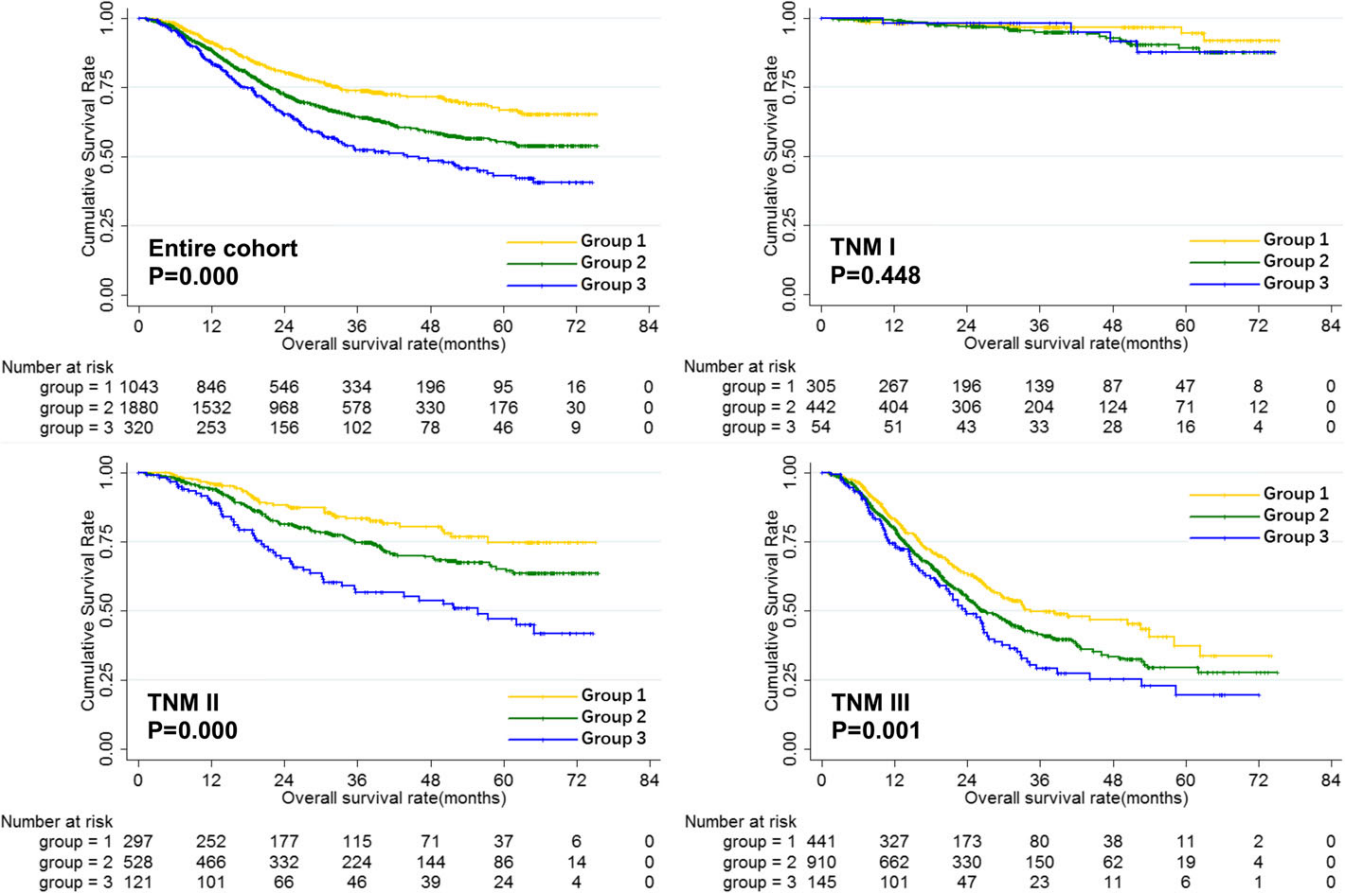
Overall survival of gastric cancer patients according to the combination of lymphocyte and monocyte count

Further, the prognostic value of combination of lymphocyte and monocyte for gastric cancer patients were evaluated. Patients were divided into three groups according to the levels of lymphocyte and monocyte: Group 1: patients with high lymphocyte and low monocyte; Group 2: patients with high lymphocyte and high monocyte, or low lymphocyte and low monocyte; Group 3: patients with low lymphocyte and high monocyte. As the prognosis of patients with high lymphocyte and high monocyte and that with low lymphocyte and low monocyte were comparable, these patients were all assigned to group 2. The overall survival of the three groups were shown in Fig. 4. The results showed that combination of lymphocyte and monocyte count could increase the predictive value for the prognosis of the entire cohort. Moreover, we found that combination of lymphocyte and monocyte count could only predict the prognosis of stage II/III gastric cancer patients, but not stage I gastric cancer patients.

## Discussion

Most studies focused on the prognostic value of NLR and PLR in gastric cancer patients. Full analysis of the prognostic value of absolute count of blood cells in gastric cancer was lacking. Therefore, the present study investigated the prognostic value of absolute count of WBC, neutrophil, lymphocyte, monocyte and platelet in gastric cancer patients. We found that high absolute count of neutrophil, monocyte and platelet and low absolute count of lymphocyte were associated with poor prognosis of gastric cancer. However, only lymphocyte and monocyte count were independent prognostic predictors. Moreover, combination of lymphocyte and monocyte count could further increase the predictive value for the prognosis of stage II/III gastric cancer patients but not stage I patients.

Eo et al. reported that absolute lymphocyte count and monocyte count were associated with the disease-free survival and overall survival of gastric cancer patients [6]. However, the two parameters were not independent prognostic factors. This may attribute to the relatively small sample size in the study. In a study containing 250 cases of surgically treated gastric cancer patients, Heras et al. found that increase of platelet count was correlated with tumor progression and unfavorable prognosis of gastric cancer [7]. Zhang et al. also reported that elevated platelet count was associated with poor prognosis in patients with gastric cancer [1]. In our present study, which contained a relatively large sample size of 3243 cases, neutrophil, lymphocyte, monocyte and platelet were all associated with prognosis of gastric cancer patients, and lymphocyte and monocyte were independent risk factors.

Neutrophil is one of the inflammatory markers [8]. Neutrophils could promote growth and metastasis of tumors through secreting a variety of cytokines, including matrix metalloproteinase-9 [9], chemokines [10] and vascular endothelial growth factor (VEGF) [11]. It was reported that neutrophils could promote adhesion between circulating tumor cells and distant target organs through acting as an adhesive adapter, finally increasing the chance of distant metastasis [12]. Moreover, neutrophil could also inhibit the antitumor immune function of natural killer cells and cytotoxic T cells [13].

Lymphocyte plays prominent role in the tumor related immunology. It possesses potent antitumor immune function that could inhibit progression of several tumors [14], and elevated level of lymphocyte was reported to be associated with favorable prognosis of a variety of tumors [15]. It was also reported that several subtypes of tumor infiltrating lymphocyte were associated with better outcomes of a variety of tumors [16–18], including CD8+ T cells [19] and memory T cells [20]. However, some subsets of T cells were associated with progression and unfavorable prognosis of tumors, such as regulatory T cells [21] and Th17 cells [22]. Although different subset of T cells was associated with adverse prognosis of tumors, high level of absolute lymphocyte count was demonstrated to be associated with favorable prognosis of gastric cancer patients in our present study.

Elevated monocyte was reported to be associated with the poor prognosis of a variety of tumor, including prostate cancer [23], cervical cancer [24] and hepatocellular carcinoma [25]. Monocyte could promote tumorigenesis and angiogenesis, and could also inhibit the antitumor immune response in vivo [26]. Moreover, monocytes could differentiate into tumor associated macrophages (TAM) by tumor microenvironment [27]. TAM could promote tumor angiogenesis and tumor growth through secretion of tumor necrosis factor alpha [28] and VEGF [29]. TAM could also facilitate invasion and migration of tumor cells through secreting various proteases and protease activators which could degrade extracellular matrix [29].

Platelet also plays prominent role in the tumor related inflammation [30] and thrombocytosis has been reported to be associated with poor prognosis in gastric cancer patients [7]. It is accepted that tumor cells could be damaged by mechanical trauma and shear force when passing through the microvasculature, and by the immune system in the blood stream. However, platelet could protect tumor cells against these damages through covering tumor cells [31]. Platelet could promote tumor growth by increasing angiogenesis via VEGF [32], and the association between serum VEGF level and platelet count has been demonstrated [33].

There were several limitations in the present study. Firstly, it was a retrospective analysis with relatively limited sample size. Multi-center studies are needed to verify the prognostic value of these blood cells. Secondly, the cut off value could be calculated through different methods, including median value, receiver operating characteristic curve and X-tile software. The prognostic value of blood cells based on different cut off values through different methods were not compared. Thirdly, the prognostic value of blood cells after surgery was not evaluated. Fourthly, data about recurrence were not available. As a result, the correlation between blood cell count and disease-free survival were not analyzed. Fifthly, blood cell count could not predict the prognosis of stage I gastric cancer patients in our study. The reasons were not deeply discussed. One of the reasons may be that the sample size of stage I patients was not large enough and the follow-up time was relatively short.

## Conclusions

Absolute count of blood cells was more convenient in predicting the prognosis of gastric cancer patients. High absolute count of neutrophil, monocyte and platelet, and low absolute count of lymphocyte were associated with poor prognosis of gastric cancer. However, only lymphocyte and monocyte count were independent prognostic predictors. Combination of lymphocyte and monocyte count could further increase the predictive value for gastric cancer.

## Abbreviations

NLR: Neutrophil-to-lymphocyte ratio
PLR: Platelet-to-lymphocyte ratio
TAM: Tumor associated macrophages
VEGF: Vascular endothelial growth factor
WBC: White blood cell
